# Will the reformed Cancer Drugs Fund address the most common types of uncertainty? An analysis of NICE cancer drug appraisals

**DOI:** 10.1186/s12913-018-3162-2

**Published:** 2018-05-31

**Authors:** Liz Morrell, Sarah Wordsworth, Anna Schuh, Mark R. Middleton, Sian Rees, Richard W. Barker

**Affiliations:** 1 Oxford-UCL Centre for the Advancement of Sustainable Medical Innovation, Radcliffe Department of Medicine, University of Oxford, Room 4403, Level 4, John Radcliffe Hospital, Headley Way, Headington, Oxford, OX3 9DU UK; 2 Health Economics Research Centre, Nuffield Department of Population Health, University of Oxford, Old Road Campus, Roosevelt Drive, Headington, Oxford, OX3 7LF UK; 3 Oxford NIHR Biomedical Research Centre, University of Oxford, Oxford, UK; 4 Department of Oncology, University of Oxford, Old Road Campus Research Building, Roosevelt Drive, Headington, Oxford, OX3 7DQ UK; 5 Health Experiences Institute, Nuffield Department of Primary Care Health Sciences, University of Oxford, 23-38 Hythe Bridge Street, Oxford, OX1 2ET UK

**Keywords:** Health technology appraisal, Cancer, Uncertainty, Cost-effectiveness, NICE, Cancer drugs fund

## Abstract

**Background:**

One of the functions of the reformed Cancer Drugs Fund in England is as a managed access fund, providing conditional funding for cancer drugs where there is uncertainty in the economic case, and where that uncertainty can be addressed by data collection during two years’ use in the NHS. Our study characterises likely sources of such uncertainty, through a review of recent NICE Technology Appraisals.

**Methods:**

Discussions of uncertainty in NICE Appraisal Committees were extracted from published Single Technology Appraisals of cancer drugs, 2014–2016, and categorised inductively. The location of the comments within the structured Appraisal document was used as a proxy for the degree of concern shown by the Committee.

**Results:**

Twenty-nine appraisals were analysed, of which 23 (79%) were recommended for funding. Six main sources of uncertainty were identified. Immaturity of survival data, and issues relating to comparators, were common sources of uncertainty regardless of degree of concern. Uncertainties relating to quality of life, and the patient population in the trial, were discussed frequently but rarely occurred in the more uncertain appraisals. Concerns with trial design, and cost uncertainty, were less common, but a high proportion contributed to the most uncertain appraisals. Funding decisions were not driven by uncertainty in the evidence base, but by the expected cost per QALY relative to acceptance thresholds, and the resultant level of uncertainty in the decision.

**Conclusions:**

The reformed CDF is an improvement on its predecessor. However the main types of uncertainty seen in recent cancer appraisals will not readily be resolved solely by 2 years’ RWD collection in the reformed CDF; where there are no ongoing trials to provide longer-term data, randomised trials rather than RWD may be needed to fully resolve questions of relative efficacy. Other types of uncertainty, and concerns with generalisability, may be more amenable to the RWD approach, and it is these that we expect to be the focus of data collection arrangements in the reformed CDF.

## Background

The Cancer Drugs Fund (CDF) in England was established in 2010, with the aim of improving access to new cancer drugs, by providing funding for drugs not - or not yet - recommended by the National Institute of Health and Care Excellence (NICE) for reasons of cost-effectiveness. The Fund was intended as a temporary measure until a value-based pricing approach could be introduced. This entailed broadening the scope of NICE’s appraisal process to include additional elements of ‘value’, which would be taken into account in negotiating a drug’s price, and was expected to lead to more drugs being deemed sufficiently cost-effective for NHS use [1]. However, the proposals failed to find broad stakeholder agreement, and were shelved; the CDF continued with expenditure rising from £200million at its inception in 2011/12, to over £400million in 2014/15 [2].

There has been extensive debate about the CDF’s justification [3, 4] sustainability [2] and decision processes [5], and in 2016 it underwent reform, under which all funding decisions were re-integrated into NICE. In addition to providing interim funding for newly approved cancer drugs, the reformed CDF functions as a managed access fund. Specifically, where there is uncertainty in the clinical and cost-effectiveness data such that a drug cannot be recommended for routine commissioning, it can be recommended for funding through the CDF, providing:

*1) 'The incremental cost-effectiveness ratios (ICERs) presented have the plausible potential for satisfying the criteria for routine use […]; 2) It is possible that the clinical uncertainty can be addressed through collection of outcome data from patients treated in the NHS; and 3) It is possible that the data collected (including from research already underway) will be able to inform a subsequent update of the guidance. This will normally happen within 24 months'* [6].

The challenge of uncertainty in economic evaluation is not new, and there are existing examples of conditional funding arrangements in the UK: for example, the Multiple Sclerosis Risk-Sharing Scheme [7] and more recently, managed entry schemes used for drugs for rare conditions such as Duchenne muscular dystrophy [8]. Elsewhere in Europe, examples include a coverage-with-evidence-development route in the Netherlands [9] and a series of monitoring registries in Italy [10, 11].

Given that the issue of uncertainty in economic evidence is central to the new policy, it is important to understand the uncertainties that might be expected to arise in candidates for CDF conditional funding, hence the data required to reduce that uncertainty, and the appropriate study designs for that data collection. The aim of our study was to identify likely sources of uncertainty, through undertaking a review of NICE Technology Appraisals of cancer drugs in the years immediately preceding the reform.

## Methods

Our study is based on the expectation that the uncertainties likely to be encountered by NICE in future cancer drug evaluations, will be similar to those seen in submissions in the recent past, and will undergo similar review and debate in the NICE Appraisal Committees. We therefore chose as our source, NICE’s Technology Appraisal documents (Final Appraisal Document, FAD), which publish the deliberations of the Committee and the evidence on which those discussions are based; that is, our analysis reflects evaluation of uncertainty from the perspective of a decision-making committee. A time period of the 2 complete calendar years prior to the CDF reform proposals (March 2016) was chosen, to balance recency with sufficient number of cases. FADs for cancer drugs for the period January 2014–March 2016 were accessed via the NICE website.

FADs follow a consistent template, within which the Committee’s deliberations are discussed in Section 4, ‘Consideration of the Evidence’, and this Section contains a summary table of the Committee’s key conclusions. Comments on uncertainty in these tables (both clinical and cost-effectiveness uncertainty) were extracted, tabulated in Excel, and classified using an inductive process – that is, the categories were suggested by the text rather than an imposed framework. A given piece of text was classified unambiguously (linked to only one category). We focus on the explicit meaning, and minimal inference is required in categorising; for example, discussion of Kaplan-Meier curves is related unambiguously to survival analysis. Data extraction and classification was done by LM and reviewed by SW; both are health economists familiar with this technical vocabulary. We report the prevalence of each class of uncertainty as the number of FADs in which it occurs; whilst there have been debates on the use of counts in content analysis, in this case counts are valid as described by Hannah and Lausch [12] where the counted unit is clearly defined (the FAD), and differences in occurrence rates are readily interpretable. Other TA information was also extracted, including the funding decision, and the Incremental Cost Effectiveness Ratio (ICER).

From initial familiarisation with the FADs, we noted two particular locations where uncertainty was specifically discussed. Firstly, there are two rows in the summary table which deal with uncertainty, in the clinical effectiveness and cost-effectiveness evidence respectively. Secondly, the headline of the table (which reflects key features of the appraisal overall) in some cases mentions uncertainty. To allow exploration of the strength of concern with uncertainty, we assume that the uncertainty rows of the table pick out the uncertainty issues of most concern during the Committee’s deliberations, and further that the FADs with comments on uncertainty in the headline are those where uncertainty was a highly salient feature of the decision. The prevalence of the various sources of uncertainty was then considered over three levels, treating location as a proxy for increasing concern:

- All appraisals, any comment on uncertainty in the summary table
- All appraisals, comments from the specific uncertainty sections in the table
- Only appraisals where uncertainty is specifically discussed in the headline summary section. These are referred to as ‘highly uncertain’ in our analysis.

## Results

Thirty-three appraisals were published in the specified timeframe, of which four were terminated by NICE following non-submission of data by the manufacturer, leaving 29 cases for analysis (21 solid tumours, eight haematological malignancies). Of these, 18 were recommended for funding, five optimised (that is, recommended with restrictions relative to the licenced indication) and the remaining six not recommended for funding.

We found that uncertainties in the evidence base were clearly signalled in the FADs by use of the word ‘uncertainty’, or terms such as: risk of bias, unreliable, weak, immature, not generalisable. All 29 TAs discussed uncertainty. The sources of uncertainty identified are shown in Table 1. Uncertainties in survival data and comparators are common across all levels of uncertainty. Quality of Life (QoL) data and patient population are frequently discussed, but are not as prevalent in the ‘highly uncertain’ appraisals. Cost estimates and trial design are less commonly discussed, but around half of the instances are found in the more uncertain appraisals.

**Table 1:**
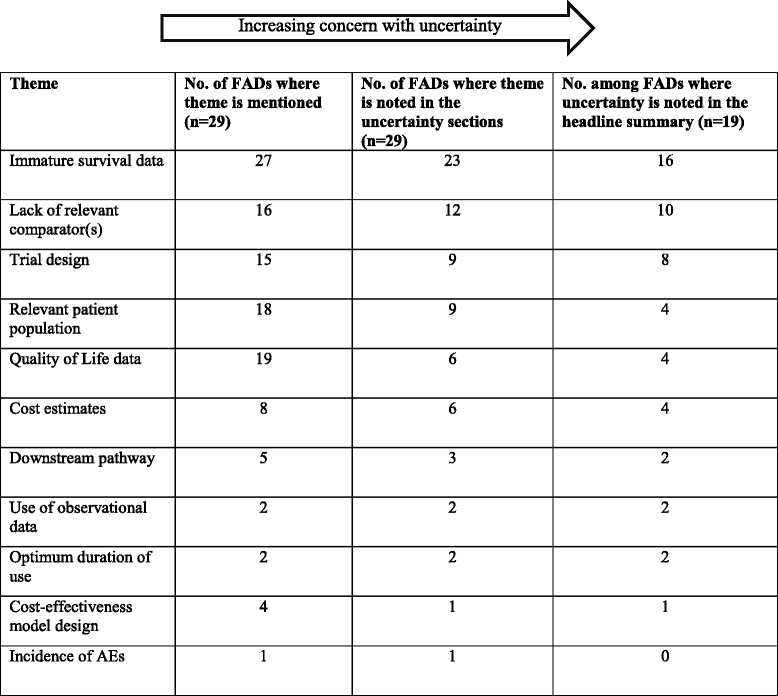
Analysis of comments on uncertainty in NICE appraisals of cancer drugs

FAD: Final Appraisal Document AEs: adverse events

FADs published January 2014–March 2016

Table 2 compares the appraisal decisions with level of uncertainty as defined. The decisions do not appear to reflect the level of uncertainty in the evidence base. Rather, they reflect the estimated value of the ICER or ICER range, relative to the relevant cost-effectiveness threshold. Drugs could be Recommended despite wide-ranging ICER estimates, if those ICERs were expected to fall within acceptable ranges; in such cases the uncertainty in the decision was low despite uncertainty in the evidence base. For example:

*‘The Committee accepted that this ICER was associated with uncertainty but, on balance, it was satisfied that it would remain below £30,000 per QALY gained*’ (enzalutamide in prostate cancer [13])

Submissions that had ICER estimates above the acceptable range were Not Recommended, regardless of the level of uncertainty (for example pomalidomide in multiple myeloma (MM) – all ICERs were above £50,000/QALY [14]).

**Table 2:** Effect of the level of uncertainty on funding decisions

	Level of uncertainty	
Decision	‘High’^a^ (*n* = 19)	‘Low’^b^ (*n* = 10)
Recommended	11	7
Optimised	4	1
Not recommended	4	2

^a^High uncertainty: appraisals where uncertainty was discussed in the summary table headline

^b^Low uncertainty: all other evaluated appraisals

We find no relationship between decision and either the specific source of uncertainty, or the number of different sources of uncertainty. For example, radium-223 dichloride (prostate cancer) was recommended in a specific subgroup despite multiple uncertainties (ICER expected to be within acceptable range) [15], and afatinib (non-small cell lung cancer, NSCLC) was recommended by analogy to similar drugs despite no ICER being calculable [16]. In contrast, trastuzumab emtansine (breast cancer) was rejected with low uncertainty across multiple areas but a high ICER (£167,000/QALY) [17], whilst ramucirumab (gastric cancer) and pemetrexed (NSCLC) were rejected with significant uncertainty each in one particular area, also with high ICERs (£188,000–408,000/QALY, £75,000/QALY respectively) [18, 19]. The main quantitative indicator of the uncertainty of the decision, used in almost all the TAs, was the probability of the technology being cost-effective at the relevant cost-effectiveness threshold.

The most common types of uncertainty are described below, with illustrative examples from the reviewed appraisals.

### Immature survival data

The uncertainty in these cases refers to a common situation in oncology, where the trial data intended to establish the relative treatment effect of a new drug, extend over a period that is short relative to patients’ long-term survival; this ‘lifetime’ horizon is used in cost-effectiveness modelling in order to capture all the health effects for the QALY estimate [20]. For example, the aflibercept (metastatic colorectal cancer) submission was based on a median follow-up of 2 years, with uncertainty arising in the extrapolation out to 15 years [21]. This issue is particularly relevant to overall survival; the cost-effectiveness models in the appraisals were typically some variant on a basic three-state model: progression-free, progressed disease, and death, hence key measures are progression-free survival (PFS) and overall survival (OS). In the cases reviewed, generally a sufficiently large proportion of the cohort had progressed, such that there is a reasonable degree of certainty in the PFS. However, a smaller number will have died; for example, in the trial of bortezomib in mantle cell lymphoma, more than half of the patients in the trial were still alive at the time of analysis, so a median OS could not be calculated [22]. There is therefore uncertainty in the OS due to the need to extrapolate, with extensive debate on the appropriate statistical method chosen for the extrapolation, as different models give rise to different projections for survival. Extrapolation of overall survival can be further complicated by the other uncertainties, such as duration of treatment (described under section, Cost); if the optimum duration of treatment is not known it becomes challenging to estimate the associated survival, as in the appraisal of nivolumab in advanced melanoma [23].

Although PFS is a common surrogate outcome for OS, one example (bortezomib induction therapy in MM) also directly used response rate; the aim of treatment is to allow more patients to proceed to stem cell transplant, and it was considered plausible that the observed effect of bortezomib on response rate could be associated with improved overall survival [24]. In the case of the erythropoiesis-stimulating agents, extending survival is not the primary effect of treatment; the benefit is in avoiding the cost and disutility of blood transfusion, which is sufficient to generate an acceptable ICER [25].

### Lack of relevant comparator(s)

In these examples, the drugs had not been tested directly against the treatment(s) considered to be the relevant comparator(s) in England, as defined in the respective FAD. Examples included trials against alternatives no longer in routine use in England due to practice changes since the trials were done (9/16; for example, comparison of melanoma drugs against dacarbazine, which is now rarely used [26]), or against placebo or best supportive care where an active treatment is now used (2/16 cases; for example comparison with best supportive care in prostate cancer where docetaxel or abiraterone is now used [15]). In three cases, practice across England was described as highly variable, resulting in a large number of potential comparators which would have been infeasible to include in trials (eg pomalidomide in MM at third or subsequent relapse [14]). In the appraisals, the relative effectiveness of the drugs is estimated using indirect treatment comparison or network meta-analysis.

### Trial design

The most common examples here were treatment divergences from licenced or current practice: use of doses (eg pembrolizumab in melanoma [27]) or regimens (eg nintedanib in NSCLC [28]) that differ from the licence, or use of a different dose form (eg bortezomib in MM – intravenous vs sub-cutaneous [24]). Use of crossover from the control arm in the trial design was also important, creating difficulties in attributing ultimate survival to the effect of a specific trial drug. A similar effect is created by the effects of other drugs used later in the treatment pathway, which was a source of uncertainty found infrequently in this study (Table 1). Other trial effects include small or single arm studies (eg idelalisib in untreated chronic lymphocytic leukaemia, CLL [29]), and debate on methods for establishing progression (eg olaparib in ovarian cancer [30]).

### Population in the trials

These are examples where the patient sample in the trials does not precisely match the licenced indication (eg pembrolizumab in melanoma: prior drug exposure [31]), or the restricted indication under consideration in the TA; for example, a drug may be restricted to patients with a specific mutation (eg erlotinib in NSCLC: EGFR-TK mutation [32]), to a specific line of treatment (eg olaparib in ovarian cancer: 4th or later line [30]) or a particular tumour types (eg nintedanib: NSCLC with adenocarcinoma histology [28]). The evaluation is therefore being performed using a post hoc secondary analysis of the trial data. There were further cases where the trial locations did not include the UK (eg afatinib in NSCLC: trials in an Asian population [16]) leading to concerns with generalisability to a British population if there is reasonable expectation of racial or geographic differences. In addition a small number of the appraisals comment on the general concern that trial patients tend to be younger and fitter than the treated population.

### Quality of life data

Issues in this area are typically absence of health-related Quality of Life (QoL) data – not collected in the trials, collected using a non-comparable tool (eg ipilimumab in melanoma: EORTC-CRC30 rather than EQ5D [33]), collected but not used in the submission (eg obinutuzumab in CLL [34]). In these situations, values from the literature or alternative methods are used, and these have varying levels of validity. In one further case, health-related QoL was valued using a non-UK value set (paclitaxel in pancreatic cancer: US value set for EQ5D [35]); this creates debate but can readily be converted using the raw data.

### Cost

The uncertainties in cost are varied in their reasons. Uncertainties in survival on treatment, and optimal duration of treatment, led to important uncertainties in drug cost (eg pembrolizumab in previously untreated melanoma [27]). This uncertainty is not mitigated by Patient Access Schemes based on dose capping, because the average cost of the drug then becomes uncertain (eg lenalidomide in myelodysplastic syndrome: no drug cost to NHS after 26 cycles [36]). Other cost uncertainties include costs of treating adverse events, and the impact of vial sharing and dose reduction.

## Discussion

Common sources of uncertainty in technology appraisals of cancer drugs during 2014–15 are the overall survival estimates, and availability of relevant comparator data. Other sources of uncertainty are Quality of Life data, trial design, patient population, and costs. These findings are consistent with informal comments from current and former committee members, and our observation of NICE Appraisal Committee meetings from the public gallery. Funding decisions do not appear to be driven by the level or types of uncertainty *per se*, but by expected cost-effectiveness relative to the cost-effectiveness threshold.

Neither of the two main types of uncertainty is likely to be readily resolved by generating 2 years of ‘real world’ data (RWD) within the CDF. There are difficulties with use of observational data to generate evidence of relative effectiveness, as described by Grieve et al. [37]. In a randomised trial, randomisation allows outcomes to be compared between groups of patients who differ in treatment received, but are similar in other regards; in contrast, in clinical use, patients are prescribed a given therapy based on clinical characteristics, and are therefore systematically different from patients on any other treatment, thus introducing confounding into a between-treatment comparison. Grieve et al. propose wider use of ‘only in research’ recommendations, to support pragmatic randomised trials within the NHS [37]. Our findings support this suggestion in situations where the uncertainty concerns the relative treatment effect, and RWD carries the risk of selection bias.

The issue of confounding is highly relevant to uncertainty relating to comparators, as the patients on the comparator treatment in real-world use will be different from those prescribed the drug of interest. Options for establishing baseline survival on the current regimens could include historical data from real-world use (such as registry data prior to introduction of the new treatment), other trial data, or in-use data from other countries; such sources need careful consideration for their generalisability to the current, UK, clinical population. For example, in the recent appraisal of avelumab in metastatic Merkel cell carcinoma, survival data for current standard of care was provided by an observational study; however no further comparator data can now be generated as avelumab is changing that standard of care globally [38].

For survival uncertainty, in addition to concerns with using RWD, we also need to consider the timeframe. In examples from this dataset, the submitted trials already have 2+ years’ data, and yet there remains substantial uncertainty in OS; 2 years’ *de novo* in-use data will provide no new information on long-term survival. In this situation, the role of CDF funding is to allow clinical use whilst the original survival data matures in ongoing trials. This supports access to promising drugs showing a marked improvement in survival, where the uncertainty paradoxically derives from the resulting low number of events (progression or death) during the trials. However, there may be specific situations where 2 years’ RWD could contribute to uncertainty reduction, such as where existing trials are small, or demonstrating a relationship between survival and a surrogate marker.

Uncertainty due to drug regimens used in trials, small sample size, patient population differences, QoL data, incidence of AEs, and cost, may be more amenable to resolution through RWD; for example, measuring duration of use or dosage patterns, where a comparison is not required, or where the data are to verify consistency with predictions from another population or from a model. It is these types of uncertainty that we might expect to see leading to conditional funding through the reformed CDF, and review of CDF entrants illustrates that this has been the case; the majority involve survival data from ongoing trials, with RWD collection predominantly on treatment patterns, and for generalisability. For example, the first drug to be funded through the new CDF was osimertinib in NSCLC. The appraisal identifies uncertainty in the overall survival extrapolation, and the generalisability of the trial data to UK clinical practice. The data collection arrangements define future analyses of the ongoing trials to resolve the survival uncertainty, and focus the RWD collection on duration of treatment and baseline characteristics of the patient population [39]. A more recent example (atezolizumab in urothelial carcinoma) similarly relies on an ongoing Phase III trial for survival data, with NHS data collection on treatment duration [40]. Of note, the practice of linking the review of an appraisal to updates of the clinical data was evident before implementation of the reformed CDF; for example, nivolumab in melanoma has a scheduled review to coincide with updated survival data and studies on optimal treatment duration [23].

Uncertainty in economic evaluation is typically described as four types [41, 42], summarised in Table 3. In our analysis the main types of uncertainty observed were generalisability (patient populations), or related to the assumptions and choices made in the estimates, which can be described as structural uncertainty (survival extrapolation modelling, indirect and mixed treatment comparisons). These are overlaid on inherent parameter uncertainty, which can be characterised effectively using well established methods of probabilistic sensitivity analysis (PSA) and value of information analysis [42, 43]. In contrast, structural uncertainty and generalisability have been less studied [44, 45] and are typically handled by one-way scenario analysis. Whilst this gives an indication of the impact of specific alternative assumptions, it cannot fully characterise the complex interactions of the various sources of uncertainty without computing large numbers of alternatives, and provides no indication of the likelihood of a given result, leading to high uncertainty of decision-making. Recent papers recommend parameterising the uncertainty so it is reflected in the cost-effectiveness model and hence in the PSA [44, 46, 47], with Sculpher et al. [47] providing examples. Further development of relevant methods or other decision support tools may be helpful for decision-makers faced with this type of uncertainty.

**Table 3:** Taxonomy of types of uncertainty in cost-effectiveness models

Type of uncertainty	Description	Handled by:
Parameter uncertainty	Uncertainty in estimates of the values of the parameters used in the cost-effectiveness model, represented by the familiar concepts of standard deviation and standard error	Probabilistic sensitivity analysis
Structural uncertainty	The assumptions made in constructing and populating cost-effectiveness models, such as the method used to extrapolate survival	Sensitivity analysis
Methodological uncertainty	The analytical approaches used	Specification of a Reference Case of standard methods
Generalisability	To what extent the model, assumptions and data represent the population for which the decision is being made	Sensitivity analysis

The reformed CDF goes part of the way towards the proposals of Buxton et al. [3], in giving the CDF a specific role in addressing issues of uncertainty, and in re-integrating all cancer drug funding decisions under NICE rather than providing an alternative funding stream. Further, the fund’s pricing requirements provide a mechanism for NICE to recommend drugs that might otherwise have been rejected, thus providing health benefit for the population at a cost-effective price. These features are improvements on the original CDF. The reforms stop short, however, of proposing additional data collection through new randomised trials, relying on RWD and ongoing trials, and this has been criticised as a missed opportunity to generate new, robust data for decision-making [37].

There is potential for improvement in approaches to RWD. The UK in principle is well placed to generate routine data; collection is centralised - described as the largest of its type at its inception in 2013, with data extending over 30 years - and reporting to the Systemic Anti-Cancer Therapy dataset has been mandatory since 2014. This capability enables NHS data collection in the CDF managed access agreements. There is however a need for clear frameworks for integrating such data with trial evidence, and how the results will be used in decision-making when compared to cost-effectiveness standards based on RCTs. This could helpfully include direction on statistical methods for accounting for selection bias. Further, it would not be unreasonable to engage the broad range of stakeholders – including patients – in design and use of RWD, in the same way as for RCTs. The GetReal project – a European cross-stakeholder consortium – made similar observations in their recommendations for improving the use of real-world evidence [48].

Uncertainty in the evidence on overall survival is inevitable, when trials are short relative to long-term survival. With current initiatives on earlier access (for example, the Accelerated Access Review and the UK’s Early Access to Medicines Scheme), the challenges of dealing with uncertainty are likely to increase, with growing reliance on surrogate outcomes [49]. Although surrogate outcomes have the advantage of providing results more quickly, systematic reviews suggest that the correlation between surrogates and OS in cancer is generally low [50, 51], although stronger in some specific examples (for example PFS in advanced colorectal cancer [50]). Hence validation of the relationship is essential, and that validation is specific to the tumour type, treatment, and treatment setting [52]. Importantly though, in the context of cost-effectiveness, the surrogate outcome of PFS can have value in itself, potentially adding QALY’s by extending time in a health state with high quality of life, and that time can be highly valued by patients [53].

Beyond estimation of the probability that a technology is cost-effective, we found little evidence of use of value-of-information analyses to support decisions. Such analysis is recommended in current frameworks for handling uncertainty in decision-making [46, 47, 54]. These frameworks are outlined in NICE’s Methods guide ([20] Section 6.4), so it is perhaps surprising not to see more discussion of these concepts in the FADs. Sculpher et al. [47] discuss possible barriers, and suggest that the approaches could be used qualitatively in the absence of formal analysis. It may be that NICEs committees are considering these issues implicitly rather than using this explicit terminology, so are not reported as such in the documents. Further, with the option of conditional reimbursement through the CDF, we may see more use of such frameworks to guide decisions on the type and design of further data collection.

This study is limited by the relatively small number of appraisals included, and the secondary nature of the source, which has undergone condensation and inevitable filtering to produce the FAD. The FADs are produced by NICE with the Committee chair and are reviewed by Committee members; however there remains some risk of inconsistent reporting between Committees. Using primary transcripts would avoid this risk, but require a higher level of interpretation by the researchers. Secondly, most of the data extraction and classification was done by a single analyst. However, we were focusing on explicit content expressed in specific technical terms, rather than requiring high levels of interpretation as in, for example, thematic analysis of focus groups, where more than one researcher would code and interpret themes. Our work could be supplemented by formal interviews with committee members and NICE staff. Finally, our study focused on the reported discussions of the NICE Appraisal Committees. Hence we do not address broader issues such as global clinical trial strategies, or the ability of current Quality of Life tools to capture the full range of patient experience; these were not discussed in the appraisals we reviewed, but clearly affect the availability and quality of data for decision-making.

## Conclusion

The reformed CDF is an improvement on its predecessor. However, the main types of uncertainty seen in recent cancer appraisals relate to overall survival estimates and availability of relevant comparator data. These will not readily be resolved solely by 2 years’ RWD collection in the reformed CDF; where there are no ongoing trials to provide longer-term data, randomised trials rather than RWD may be needed to fully resolve questions of relative efficacy. Other types of uncertainty, and concerns with generalisability, may be more amenable to the RWD approach, and it is these that we expect to be the focus of data collection arrangements in the reformed CDF. We recommend further work on methods for characterisation of structural uncertainty, and continued development of thinking on how observational data can be best combined with other data types in cost-effectiveness analysis.

## Abbreviations

CDF: Cancer Drugs Fund
CLL: Chronic lymphocytic leukaemia
EoL: End of life
FAD: Final Appraisal Document
ICER: Incremental cost effectiveness ratio
MM: Multiple myeloma
NHS: National Health Service
NICE: National Institute for Health and Care Excellence
NSCLC: Non-small cell lung cancer
OS: Overall survival
PAS: Patient access scheme
PFS: Progression free survival
QALY: Quality-adjusted life year
QoL: Quality of life
RWD: Real-world data
TA: Technology Appraisal
