# Correction to: Will the reformed Cancer Drugs Fund address the most common types of uncertainty? An analysis of NICE cancer drug appraisals

**DOI:** 10.1186/s12913-019-4039-8

**Published:** 2019-03-28

**Authors:** Liz Morrell, Sarah Wordsworth, Anna Schuh, Mark R. Middleton, Sian Rees, Richard W. Barker

**Affiliations:** 1 Oxford-UCL Centre for the Advancement of Sustainable Medical Innovation, Radcliffe Department of Medicine, University of Oxford, Room 4403, Level 4, John Radcliffe Hospital, Headley Way, Headington, Oxford, OX3 9DU UK; 2 Health Economics Research Centre, Nuffield Department of Population Health, University of Oxford, Old Road Campus, Roosevelt Drive, Headington, Oxford, OX3 7LF UK; 3 Oxford NIHR Biomedical Research Centre, University of Oxford, Oxford, UK; 4 Department of Oncology, University of Oxford, Old Road Campus Research Building, Roosevelt Drive, Headington, Oxford, OX3 7DQ UK; 5 Health Experiences Institute, Nuffield Department of Primary Care Health Sciences, University of Oxford, 23-38 Hythe Bridge Street, Oxford, OX1 2ET UK

**Correction to: BMC Health Serv Res (2018) 18:393**

**https://doi.org/10.1186/s12913-018-3162-2**

In the original publication of this article [1], the Acknowledgements section lacks of important funding information “This project was funded by the NIHR Oxford Biomedical Research Centre BRC”. The authors apologize for any inconvenience that it may have caused.
